# Supplementary material for: Predicting one-year outcome in first episode psychosis using machine learning
Source: PLoS One. 2019 Mar 7;14(3):e0212846. doi: 10.1371/journal.pone.0212846 (PMC6405084; doi:10.1371/journal.pone.0212846)
Supplement: S1 File — (DOCX) [file pone.0212846.s001.docx]

**Supplemental Methods**

**Validation Cohort**

The study was a 12-month prospective study of individuals with first-episode psychosis. Ethical (REC: 04/S0703/91) and research governance approval was granted before the start of the study. The study was conducted between 1 September 2006 and 31 August 2009.

Recruitment took place in National Health Service (NHS) mental health services in Glasgow and Edinburgh. All potential participants were approached for informed consent. Inclusion criteria were: (a) in-patients or out-patients with (b) first presentation to mental health services for psychosis, (c) DSM-IV criteria for schizophrenia, schizophreniform disorder, schizo-affective disorder, delusional disorder or bipolar disorder. [1] Members of the clinical teams providing their care identified individuals meeting these criteria and an invitation to participate in the research was extended by a member of the research team. Participation was voluntary and following receipt of informed and written consent participants were entered into the study.

The participant flow is illustrated below. Of the 102 participants eligible for consent, 79 were entered into the study of whom 74 were followed up at 6 months and 68 at 12 months.


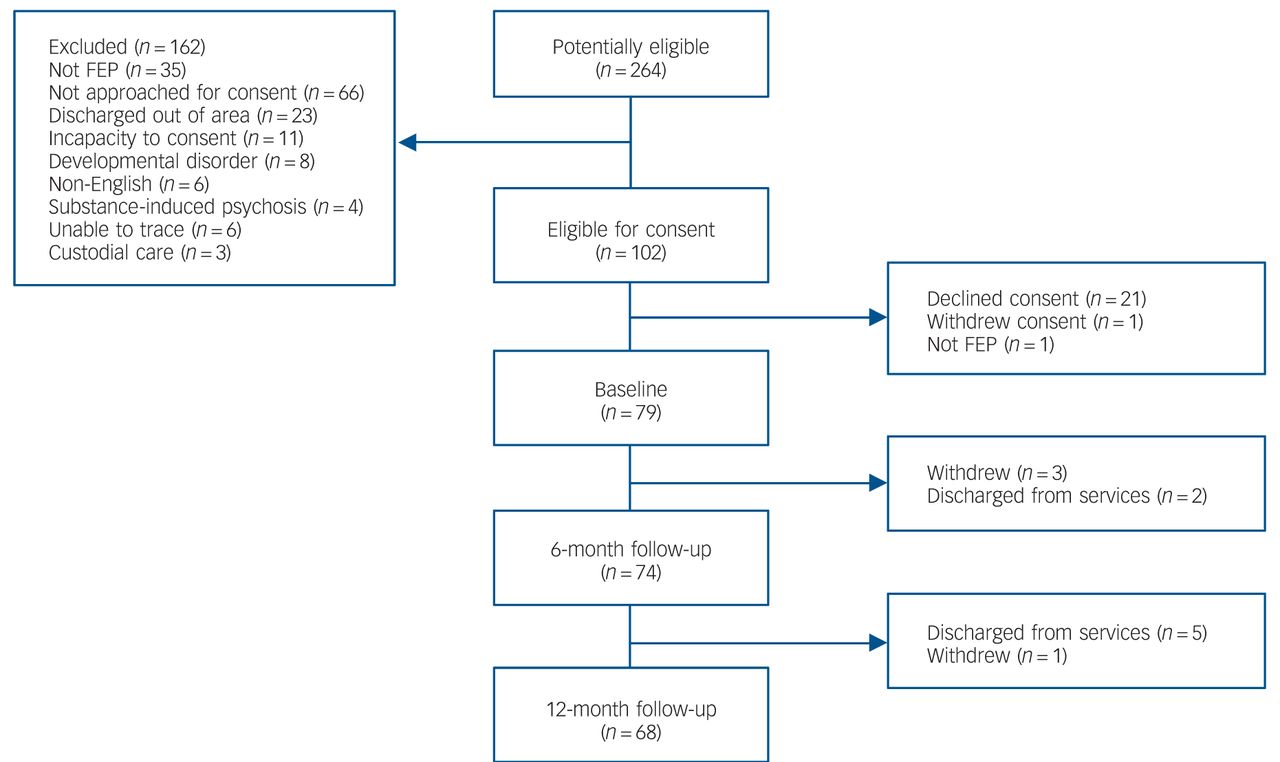
[2]

**Statistics**

Within ‘Caret’ [3], the appropriate range of lambda values for the training data was first derived by running ‘glmnet’ [4] with an alpha of 0.5. A grid search was then performed over 10 alpha values (0.1 to 1) by the 10 ‘glmnet’ derived lambda values. For each combination of alpha and lambda hyperparameters over the 10 by 10 grid, an elastic net regression model was built using 4/5 of the training set and tested on the held out 1/5 with a ROCAUC metric calculated. This was carried out 5 times by leaving 1-fold out each time. This procedure was repeated 100 times with different splits. The model with the combination of alpha and lambda hyperparameters which results in the highest average ROCAUC was selected as the best model.

**References**

1. American Psychiatric Association. Diagnostic and Statistical Manual of Mental Disorders (4th edn) (DSM-IV). APA, 1994.
2. Gumley AI, Schwannauer M, Macbeth A, et al. Insight, duration of untreated psychosis and attachment in first-episode psychosis: prospective study of psychiatric recovery over 12-month follow-up. Br J Psychiatry. 2014;205(1):60-67. doi:10.1192/bjp.bp.113.126722.
3. Kuhn M. caret: Classification and Regression Training. https://github.com/topepo/caret/.
4. Friedman J, Hastie T, Tibshirani R. Regularization Paths for Generalized Linear Models via Coordinate Descent. *J Stat Softw*. 2010;33(1):1-22. doi:10.18637/jss.v033.i01.
