# Supplementary material for: Predicting one-year outcome in first episode psychosis using machine learning
Source: PLoS One. 2019 Mar 7;14(3):e0212846. doi: 10.1371/journal.pone.0212846 (PMC6405084; doi:10.1371/journal.pone.0212846)
Supplement: S2 File — (DOCX) [file pone.0212846.s002.docx]

#load required libraries

library(readr)

library(caret)

library(doParallel)

library(foreign)

library(haven)

library(psych)

library(RANN)

library(pROC)

library(clinfun)

library(combinat)

library(gtools)

library(DescTools)

library(plyr)

#enable multicore (windows) which roughly halfs time for analysis runs !!!!WINDOWS ONLY!!!!!!!

cl <- makeCluster(detectCores(), type='PSOCK')

registerDoParallel(cl)

options(max.print=1000000)

#don't use scientific notation (revert back with options(scipen=0)

options(scipen=999)

options(digits = 4)

#load study data

preproc_all = read_csv("both_preproc.csv")

#look at the structure to work out which variables need to be changed to factors for caret

#

str(preproc_all)

preproc_all$Accommodation = as.factor(preproc_all$Accommodation)

preproc_all$Admitted_Hosp = as.factor(preproc_all$Admitted_Hosp)

preproc_all$Alcohol = as.factor(preproc_all$Alcohol)

preproc_all$Citizenship = as.factor(preproc_all$Citizenship)

preproc_all$Depression_Severity = as.factor(preproc_all$Depression_Severity)

preproc_all$Drugs = as.factor(preproc_all$Drugs)

preproc_all$Education = as.factor(preproc_all$Education)

preproc_all$Ethnicity = as.factor(preproc_all$Ethnicity)

preproc_all$Gender = as.factor(preproc_all$Gender)

preproc_all$Household = as.factor(preproc_all$Household)

preproc_all$M0_Emp = as.factor(preproc_all$M0_Emp)

preproc_all$Parent = as.factor(preproc_all$Parent)

preproc_all$Relationship = as.factor(preproc_all$Relationship)

preproc_all$M6_Emp = as.factor(preproc_all$M6_Emp)

preproc_all$M6_Rem = as.factor(preproc_all$M6_Rem)

preproc_all$M6_Res = as.factor(preproc_all$M6_Res)

preproc_all$Y1_Emp = as.factor(preproc_all$Y1_Emp)

preproc_all$Y1_Rem = as.factor(preproc_all$Y1_Rem)

preproc_all$Y1_Res = as.factor(preproc_all$Y1_Res)

preproc_all$Y1_Rem_6 = as.factor(preproc_all$Y1_Rem_6)

#get crisp

crisp_prep = preproc_all[ which(preproc_all$Cohort=="CRISP"), ]

#get fep

fep_prep = preproc_all[ which(preproc_all$Cohort=="FEP"), ]

#get columns I want crisp

crisp_prep_all = crisp_prep[ ,!(colnames(crisp_prep) %in% c("Cohort", "Depression_Severity","Education","M6_PANSS_Total_score","M6_Rem","M6_Res","M6_Emp","Y1_PANSS_Total_score"))]

#get columns I want fep

fep_prep_all = fep_prep[ ,!(colnames(fep_prep) %in% c("Cohort", "Depression_Severity","Education","M6_PANSS_Total_score","M6_Rem","M6_Res","M6_Emp","Y1_PANSS_Total_score"))]

#Analyse data for demographics table using appropropriate tests (uncomment as required)

#% missing data each column e.g.

#(sum(is.na(crisp_prep_all$Alcohol))/length(crisp_prep_all$Alcohol))*100

#fisher's test (small n) or chi squared if >4 categorical variables - get count, make table of the counts in order df = as.table(rbind(c(3,0,5,5),c(0,0,0,2))) then chisq.test(df)

#count(fep_prep_all[which(!is.na(fep_prep_all$Accommodation)),], 'Accommodation')

#count(crisp_prep_all[which(!is.na(crisp_prep_all$Accommodation)),], 'Accommodation')

#df = as.table(rbind(c(2,1,1,43,4,20),c(0,0,0,30,4,48)))

#fisher.test(df)

#chisq.test(df,correct = F)

#independent two sample t-test for continuous (Welch's as unequal sample sizes)

#t.test(crisp_prep_all$Age[which(!is.na(crisp_prep_all$Age))], fep_prep_all$Age[which(!is.na(fep_prep_all$Age))], paired = F)

#set training method as cross validation and stuff to allow metric to be roc

control <- trainControl(method="repeatedcv", number=5, repeats=100, classProbs=TRUE, summaryFunction=twoClassSummary, savePredictions = T)

##

##

##Y1 Rem (for 6 months) built on crisp and tested on fep

##

#data.frame to model predictors vs Y1 remission

crisp_prep_all_Y1_Rem = crisp_prep_all[ ,!(colnames(crisp_prep_all) %in% c("Y1_Res","Y1_Rem","Y1_Emp"))]

fep_prep_all_Y1_Rem = fep_prep_all[ ,!(colnames(fep_prep_all) %in% c("Y1_Res","Y1_Emp","Y1_Rem"))]

#omit nas from outcome only

crisp_prep_all_Y1_Rem = crisp_prep_all_Y1_Rem[which(!is.na(crisp_prep_all_Y1_Rem$Y1_Rem_6)),]

fep_prep_all_Y1_Rem = fep_prep_all_Y1_Rem[which(!is.na(fep_prep_all_Y1_Rem$Y1_Rem_6)),]

#make replicable

set.seed(987)

#build glmnet model with knn imputation on CRISP data

crisp_prep_all_Y1_Rem_glm_mod <- train(Y1_Rem_6 ~ ., data=crisp_prep_all_Y1_Rem, method="glmnet", metric="ROC", tuneLength = 10, preProc = c("nzv","zv","center", "scale","knnImpute"), trControl=control, na.action = na.pass)

#Test model on FEP data applying the same preprocessing as the train (model) object

crisp_prep_all_Y1_Rem_glm_result <- predict(crisp_prep_all_Y1_Rem_glm_mod, fep_prep_all_Y1_Rem, type = "prob", na.action = na.pass)

crisp_prep_all_Y1_Rem_glm_result_roc = roc(predictor = crisp_prep_all_Y1_Rem_glm_result$Yes, response = fep_prep_all_Y1_Rem$Y1_Rem_6=="Yes")

crisp_prep_all_Y1_Rem_glm_result_auc = roc(predictor = crisp_prep_all_Y1_Rem_glm_result$Yes, response = fep_prep_all_Y1_Rem$Y1_Rem_6=="Yes")$auc

crisp_prep_all_Y1_Rem_glm_result_auc

plot(roc(predictor = crisp_prep_all_Y1_Rem_glm_result$Yes, response = fep_prep_all_Y1_Rem$Y1_Rem_6=="Yes"))

png("figure3.png",res = 300, width = 15, height = 15, units = 'cm')

plot(roc(predictor = crisp_prep_all_Y1_Rem_glm_result$Yes, response = fep_prep_all_Y1_Rem$Y1_Rem_6=="Yes"))

dev.off()

coords(roc(predictor = crisp_prep_all_Y1_Rem_glm_result$Yes, response = fep_prep_all_Y1_Rem$Y1_Rem_6=="Yes"), "best", best.method = "closest.topleft", ret = c("specificity", "sensitivity", "accuracy", "tn", "tp", "fn", "fp", "npv", "ppv"))

#permutation test of outcomes https://www.quora.com/How-is-statistical-significance-determined-for-ROC-curves-and-AUC-values

set.seed(987)

crisp_prep_all_Y1_Rem_glm_result_auc_null = NULL

for(i in seq (1:10000))

{

fep_perm = permute(fep_prep_all_Y1_Rem$Y1_Rem_6)

crisp_prep_all_Y1_Rem_glm_result_auc_null = c(crisp_prep_all_Y1_Rem_glm_result_auc_null, roc(predictor = crisp_prep_all_Y1_Rem_glm_result$Yes, response = fep_perm=="Yes")$auc)

}

MeanCI(crisp_prep_all_Y1_Rem_glm_result_auc_null)

histogram(crisp_prep_all_Y1_Rem_glm_result_auc_null)

#get p value by taking proportion of permutated values greater or equal to the actual value

#add a pseudocount https://genetrail2.bioinf.uni-sb.de/help?topic=p_value_computation

(1+sum(crisp_prep_all_Y1_Rem_glm_result_auc_null >= crisp_prep_all_Y1_Rem_glm_result_auc))/10000

#Computes the nonparametric area under the ROC curve and its variance based on U-statistic theory (DDCP).

roc.area.test(crisp_prep_all_Y1_Rem_glm_result$Yes, fep_prep_all_Y1_Rem$Y1_Rem_6=="Yes")

#95CI = 1.96*SE=1.96*S.D./sqrt(n)

(1.96*sqrt(roc.area.test(crisp_prep_all_Y1_Rem_glm_result$Yes, fep_prep_all_Y1_Rem$Y1_Rem_6=="Yes")$var)/sqrt(length(crisp_prep_all_Y1_Rem_glm_result$Yes)))

#final model coefs

coef(crisp_prep_all_Y1_Rem_glm_mod$finalModel, crisp_prep_all_Y1_Rem_glm_mod$bestTune$lambda)

#final model coefs

crisp_coef_Rem = coef(crisp_prep_all_Y1_Rem_glm_mod$finalModel, crisp_prep_all_Y1_Rem_glm_mod$bestTune$lambda)

names = dimnames(crisp_coef_Rem)[[1]]

crisp_coef_Rem = crisp_coef_Rem[-1]

names = names[-1]

crisp_coef_Rem = crisp_coef_Rem[c(1,3,11,12,16,18,30,33,36,37,38,44,49)]

names = names[c(1,3,11,12,16,18,30,33,36,37,38,44,49)]

#rename

names = c("Private Accommodation with Family", "Rented Accommodation", "Educational Attainment", "White Ethnicity", "Living with Spouse & Children", "PANSS G1 - Somatic Concern", "PANSS G6 - Depression", "PANSS G9 - Unusual Thought Content", "PANSS N3 - Poor Rapport", "PANSS N4 - Passive Social Withdrawal", "PANSS N5 - Difficulty in Abstract Thinking", "PANSS P4 - Excitement", "In a Relationship")

group = ifelse(crisp_coef_Rem>0,"+","-")

crisp_odds_ratio_Rem = exp(crisp_coef_Rem)

df = data.frame(names, crisp_coef_Rem, group, crisp_odds_ratio_Rem)

df$names <- factor(df$names, levels = df$names[order(df$crisp_odds_ratio_Rem)])

png("figure6.png", res = 300, width = 45, height = 15, units = 'cm')

ggplot(df,aes(x=names,y=crisp_odds_ratio_Rem,fill=group))+scale_y_continuous(limits = c(0.75, 1.5),

breaks = c(0.75, 1, 1.5), trans = scales::log10_trans())+geom_bar(stat="identity")+

coord_flip()+theme(legend.position="none")+xlab(NULL)+ylab("Odds Ratio")+

theme(axis.text.y = element_text(face="bold",size=12))+

geom_text(aes(label=sprintf("%0.3f", round(crisp_odds_ratio_Rem, digits = 3))),

y=ifelse(crisp_coef_Rem< 0.015 & crisp_coef_Rem > -0.015 ,crisp_coef_Rem - 0.0025, crisp_coef_Rem/5),

colour = ifelse(crisp_coef_Rem< 0.015 & crisp_coef_Rem > -0.015 ,"#F8766D", "white"), fontface = "bold")

dev.off()

##

##

##Y1 Rem (point) built on crisp and tested on fep

##

#data.frame to model predictors vs Y1 point remission

crisp_prep_all_Y1_RemP = crisp_prep_all[ ,!(colnames(crisp_prep_all) %in% c("Y1_Res","Y1_Rem_6","Y1_Emp"))]

fep_prep_all_Y1_RemP = fep_prep_all[ ,!(colnames(fep_prep_all) %in% c("Y1_Res","Y1_Emp","Y1_Rem_6"))]

#omit nas from outcome only

crisp_prep_all_Y1_RemP = crisp_prep_all_Y1_RemP[which(!is.na(crisp_prep_all_Y1_RemP$Y1_Rem)),]

fep_prep_all_Y1_RemP = fep_prep_all_Y1_RemP[which(!is.na(fep_prep_all_Y1_RemP$Y1_Rem)),]

#make replicable

set.seed(987)

#build glmnet model with knn imputation on CRISP data

crisp_prep_all_Y1_RemP_glm_mod <- train(Y1_Rem ~ ., data=crisp_prep_all_Y1_RemP, method="glmnet", metric="ROC", tuneLength = 10, preProc = c("nzv","zv","center", "scale","knnImpute"), trControl=control, na.action = na.pass)

#Test model on FEP data applying the same preprocessing as the train (model) object

crisp_prep_all_Y1_RemP_glm_result <- predict(crisp_prep_all_Y1_RemP_glm_mod, fep_prep_all_Y1_RemP, type = "prob", na.action = na.pass)

crisp_prep_all_Y1_RemP_glm_result_roc = roc(predictor = crisp_prep_all_Y1_RemP_glm_result$Yes, response = fep_prep_all_Y1_RemP$Y1_Rem=="Yes")

crisp_prep_all_Y1_RemP_glm_result_auc = roc(predictor = crisp_prep_all_Y1_RemP_glm_result$Yes, response = fep_prep_all_Y1_RemP$Y1_Rem=="Yes")$auc

crisp_prep_all_Y1_RemP_glm_result_auc

plot(roc(predictor = crisp_prep_all_Y1_RemP_glm_result$Yes, response = fep_prep_all_Y1_RemP$Y1_Rem=="Yes"))

png("figure2.png",res = 300, width = 15, height = 15, units = 'cm')

plot(roc(predictor = crisp_prep_all_Y1_RemP_glm_result$Yes, response = fep_prep_all_Y1_RemP$Y1_Rem=="Yes"))

dev.off()

coords(roc(predictor = crisp_prep_all_Y1_RemP_glm_result$Yes, response = fep_prep_all_Y1_RemP$Y1_Rem=="Yes"), "best", best.method = "closest.topleft", ret = c("specificity", "sensitivity", "accuracy", "tn", "tp", "fn", "fp", "npv", "ppv"))

#permutation test of outcomes https://www.quora.com/How-is-statistical-significance-determined-for-ROC-curves-and-AUC-values

set.seed(987)

crisp_prep_all_Y1_RemP_glm_result_auc_null = NULL

for(i in seq (1:10000))

{

fep_perm = permute(fep_prep_all_Y1_RemP$Y1_Rem)

crisp_prep_all_Y1_RemP_glm_result_auc_null = c(crisp_prep_all_Y1_RemP_glm_result_auc_null, roc(predictor = crisp_prep_all_Y1_RemP_glm_result$Yes, response = fep_perm=="Yes")$auc)

}

MeanCI(crisp_prep_all_Y1_RemP_glm_result_auc_null)

histogram(crisp_prep_all_Y1_RemP_glm_result_auc_null)

#get p value by taking proportion of permutated values greater or equal to the actual value

#add a pseudocount https://genetrail2.bioinf.uni-sb.de/help?topic=p_value_computation

(1+sum(crisp_prep_all_Y1_RemP_glm_result_auc_null >= crisp_prep_all_Y1_RemP_glm_result_auc))/10000

#Computes the nonparametric area under the ROC curve and its variance based on U-statistic theory (DDCP).

roc.area.test(crisp_prep_all_Y1_RemP_glm_result$Yes, fep_prep_all_Y1_RemP$Y1_Rem=="Yes")

#95CI = 1.96*SE=1.96*S.D./sqrt(n)

(1.96*sqrt(roc.area.test(crisp_prep_all_Y1_RemP_glm_result$Yes, fep_prep_all_Y1_RemP$Y1_Rem=="Yes")$var)/sqrt(length(crisp_prep_all_Y1_RemP_glm_result$Yes)))

#final model coefs

coef(crisp_prep_all_Y1_RemP_glm_mod$finalModel, crisp_prep_all_Y1_RemP_glm_mod$bestTune$lambda)

#final model coefs

crisp_coef_RemP = coef(crisp_prep_all_Y1_RemP_glm_mod$finalModel, crisp_prep_all_Y1_RemP_glm_mod$bestTune$lambda)

names = dimnames(crisp_coef_RemP)[[1]]

crisp_coef_RemP = crisp_coef_RemP[-1]

names = names[-1]

crisp_coef_RemP = crisp_coef_RemP[c(3,16,30,37,44)]

names = names[c(3,16,30,37,44)]

#rename

names = c("Rented Accomodation", "Living with Spouse & Children", "PANSS G6 - Depression", "PANSS N4 - Passive Social Withdrawal", "PANSS P4 - Excitement")

group = ifelse(crisp_coef_RemP>0,"+","-")

crisp_odds_ratio_RemP = exp(crisp_coef_RemP)

df = data.frame(names, crisp_coef_RemP, group, crisp_odds_ratio_RemP)

df$names <- factor(df$names, levels = df$names[order(df$crisp_odds_ratio_RemP)])

png("figure5.png", res = 300, width = 45, height = 15, units = 'cm')

ggplot(df,aes(x=names,y=crisp_odds_ratio_RemP,fill=group))+scale_y_continuous(limits = c(0.75, 1.5),breaks = c(0.75, 1, 1.5), trans = scales::log10_trans())+geom_bar(stat="identity")+coord_flip()+theme(legend.position="none")+xlab(NULL)+ylab("Odds Ratio")+theme(axis.text.y = element_text(face="bold",size=12))+geom_text(aes(label=sprintf("%0.3f", round(crisp_odds_ratio_RemP, digits = 3))), y=ifelse(crisp_coef_RemP< 0.015 & crisp_coef_RemP > -0.015 ,crisp_coef_RemP - 0.0005, crisp_coef_RemP/5), colour = ifelse(crisp_coef_RemP< 0.015 & crisp_coef_RemP > -0.015 ,"#F8766D", "white"), fontface = "bold")

dev.off()

#

#

#Y1 EET model built on crisp and tested on fep

#

#data.frame to model predictors vs Y1 EET

crisp_prep_all_Y1_Emp = crisp_prep_all[ ,!(colnames(crisp_prep_all) %in% c("Y1_Res","Y1_Rem","Y1_Rem_6"))]

fep_prep_all_Y1_Emp = fep_prep_all[ ,!(colnames(fep_prep_all) %in% c("Y1_Res","Y1_Rem","Y1_Rem_6"))]

#omit nas from outcome only

crisp_prep_all_Y1_Emp = crisp_prep_all_Y1_Emp[which(!is.na(crisp_prep_all_Y1_Emp$Y1_Emp)),]

fep_prep_all_Y1_Emp = fep_prep_all_Y1_Emp[which(!is.na(fep_prep_all_Y1_Emp$Y1_Emp)),]

#make replicable

set.seed(987)

#build glmnet model with knn imputation on CRISP data

crisp_prep_all_Y1_Emp_glm_mod <- train(Y1_Emp ~ ., data=crisp_prep_all_Y1_Emp, method="glmnet", metric="ROC", tuneLength = 10, preProc = c("nzv","zv","center", "scale","knnImpute"), trControl=control, na.action = na.pass)

#Test model on FEP data applying the same preprocessing as the train (model) object

crisp_prep_all_Y1_Emp_glm_result <- predict(crisp_prep_all_Y1_Emp_glm_mod, fep_prep_all_Y1_Emp, type = "prob", na.action = na.pass)

crisp_prep_all_Y1_Emp_glm_result_roc = roc(predictor = crisp_prep_all_Y1_Emp_glm_result$Yes, response = fep_prep_all_Y1_Emp$Y1_Emp=="Yes")

crisp_prep_all_Y1_Emp_glm_result_auc = roc(predictor = crisp_prep_all_Y1_Emp_glm_result$Yes, response = fep_prep_all_Y1_Emp$Y1_Emp=="Yes")$auc

crisp_prep_all_Y1_Emp_glm_result_auc

plot(roc(predictor = crisp_prep_all_Y1_Emp_glm_result$Yes, response = fep_prep_all_Y1_Emp$Y1_Emp=="Yes"))

png("figure1.png", res = 300, width = 15, height = 15, units = 'cm')

plot(roc(predictor = crisp_prep_all_Y1_Emp_glm_result$Yes, response = fep_prep_all_Y1_Emp$Y1_Emp=="Yes"))

dev.off()

coords(roc(predictor = crisp_prep_all_Y1_Emp_glm_result$Yes, response = fep_prep_all_Y1_Emp$Y1_Emp=="Yes"), "best", best.method = "closest.topleft", ret = c("specificity", "sensitivity", "accuracy", "tn", "tp", "fn", "fp", "npv", "ppv"))

#permutation test of outcomes https://www.quora.com/How-is-statistical-significance-determined-for-ROC-curves-and-AUC-values

set.seed(987)

crisp_prep_all_Y1_Emp_glm_result_auc_null = NULL

for(i in seq (1:10000))

{

fep_perm = permute(fep_prep_all_Y1_Emp$Y1_Emp)

crisp_prep_all_Y1_Emp_glm_result_auc_null = c(crisp_prep_all_Y1_Emp_glm_result_auc_null, roc(predictor = crisp_prep_all_Y1_Emp_glm_result$Yes, response = fep_perm=="Yes")$auc)

}

MeanCI(crisp_prep_all_Y1_Emp_glm_result_auc_null)

histogram(crisp_prep_all_Y1_Emp_glm_result_auc_null)

#get p value by taking proportion of permutated values greater or equal to the actual value

#add a pseudocount https://genetrail2.bioinf.uni-sb.de/help?topic=p_value_computation

(1+sum(crisp_prep_all_Y1_Emp_glm_result_auc_null >= crisp_prep_all_Y1_Emp_glm_result_auc))/10000

#Computes the nonparametric area under the ROC curve and its variance based on U-statistic theory (DDCP).

roc.area.test(crisp_prep_all_Y1_Emp_glm_result$Yes, fep_prep_all_Y1_Emp$Y1_Emp=="Yes")

#95CI = 1.96*SE=1.96*S.D./sqrt(n)

(1.96*sqrt(roc.area.test(crisp_prep_all_Y1_Emp_glm_result$Yes, fep_prep_all_Y1_Emp$Y1_Emp=="Yes")$var)/sqrt(length(crisp_prep_all_Y1_Emp_glm_result$Yes)))

#final model coefs

crisp_coef_Emp = coef(crisp_prep_all_Y1_Emp_glm_mod$finalModel, crisp_prep_all_Y1_Emp_glm_mod$bestTune$lambda)

names = dimnames(crisp_coef_Emp)[[1]]

crisp_coef_Emp = crisp_coef_Emp[-1]

crisp_coef_Emp = crisp_coef_Emp[c(6,11,16,17,41,46,47)]

names = names[-1]

names = names[c(6,11,16,17,41,46,47)]

#rename

names = c("Alcohol Use", "Educational Attainment", "Living with Spouse & Children", "Baseline EET", "PANSS P1 - Delusions", "PANSS P6 - Suspiciousness", "PANSS P7 - Hostility")

group = ifelse(crisp_coef_Emp>0,"+","-")

#crisp_odds_ratio_Emp = ifelse(crisp_coef_Emp>0,exp(abs(crisp_coef_Emp)),-exp(abs(crisp_coef_Emp)))

crisp_odds_ratio_Emp = exp(crisp_coef_Emp)

df = data.frame(names, crisp_coef_Emp, group,crisp_odds_ratio_Emp)

df$names <- factor(df$names, levels = df$names[order(df$crisp_odds_ratio_Emp)])

png("figure4.png", res = 300, width = 45, height = 15, units = 'cm')

ggplot(df,aes(x=names,y=crisp_odds_ratio_Emp,fill=group))+scale_y_continuous(limits = c(0.75, 1.5),breaks = c(0.75, 1, 1.5), trans = scales::log10_trans())+geom_bar(stat="identity")+coord_flip()+theme(legend.position="none")+xlab(NULL)+ylab("Odds Ratio")+theme(axis.text.y = element_text(face="bold",size=12))+geom_text(aes(label=sprintf("%0.3f", round(crisp_odds_ratio_Emp, digits = 3))), y=crisp_coef_Emp/5, colour = "white", fontface = "bold")

dev.off()

#END
